# Keratinocytes Exposed to Blue or Red Light: Proteomic Characterization Showed Cytoplasmic Thioredoxin Reductase 1 and Aldo-Keto Reductase Family 1 Member C3 Triggered Expression

**DOI:** 10.3390/ijms242216189

**Published:** 2023-11-10

**Authors:** Raffaella Lazzarini, Maria Fiorella Tartaglione, Veronica Ciarapica, Francesco Piva, Matteo Giulietti, Gianluca Fulgenzi, Margherita Martelli, Caterina Ledda, Ermanno Vitale, Marco Malavolta, Lory Santarelli, Massimo Bracci

**Affiliations:** 1 Occupational Medicine, Department of Clinical and Molecular Sciences, Polytechnic University of Marche, 60126 Ancona, Italy; m.f.tartaglione@pm.univpm.it (M.F.T.); v.ciarapica@pm.univpm.it (V.C.); m.martelli@pm.univpm.it (M.M.); m.bracci@staff.univpm.it (M.B.); 2 Department of Specialistic Clinical and Odontostomatological Sciences, Polytechnic University of Marche, 60131 Ancona, Italy; f.piva@staff.univpm.it (F.P.);; 3 Department of Clinical and Molecular Sciences Experimental Pathology, Polytechnic University of Marche, 60126 Ancona, Italy; g.fulgenzi@staff.univpm.it; 4 Section of Occupational Medicine, Department of Clinical and Experimental Medicine, University of Catania, 95124 Catania, Italy; cledda@unict.it; 5 Faculty of Medicine and Surgery, Kore University, 94100 Enna, Italy; ermanno.vitale@unikore.it; 6 Advanced Technology Center for Aging Research and Geriatric Mouse Clinic, Scientific Technological Area, IRCCS INRCA, 60121 Ancona, Italy; m.malavolta@inrca.it

**Keywords:** blue light, red light, keratinocytes, oxidative stress, AKR1C3, TXNRD1, clock genes, mitochondria

## Abstract

Several cell-signaling mechanisms are activated by visible light radiation in human keratinocytes, but the key regulatory proteins involved in this specific cellular response have not yet been identified. Human keratinocytes (HaCaT cells) were exposed to blue or red light at low or high irradiance for 3 days in cycles of 12 h of light and 12 h of dark. The cell viability, apoptotic rate and cell cycle progression were analyzed in all experimental conditions. The proteomic profile, oxidative stress and mitochondrial morphology were additionally evaluated in the HaCaT cells following exposure to high-irradiance blue or red light. Low-irradiance blue or red light exposure did not show an alteration in the cell viability, cell death or cell cycle progression. High-irradiance blue or red light reduced the cell viability, induced cell death and cell cycle G2/M arrest, increased the reactive oxygen species (ROS) and altered the mitochondrial density and morphology. The proteomic profile revealed a pivotal role of Cytoplasmic thioredoxin reductase 1 (TXNRD1) and Aldo-keto reductase family 1 member C3 (AKR1C3) in the response of the HaCaT cells to high-irradiance blue or red light exposure. Blue or red light exposure affected the viability of keratinocytes, activating a specific oxidative stress response and inducing mitochondrial dysfunction. Our results can help to address the targets for the therapeutic use of light and to develop adequate preventive strategies for skin damage. This in vitro study supports further in vivo investigations of the biological effects of light on human keratinocytes.

## 1. Introduction

Artificial light was able to induce alterations in the biological functions and circadian rhythm in humans [1,2]. The main interest of the research was focused on the effect of ultraviolet radiation [3]. Recently, the biological response to the electromagnetic radiation belonging to the visible spectrum gained the attention of the scientific community [4,5]. The interest in the potential effects of visible radiation derives from recent technological changes in artificial lighting. Incandescent bulbs were used for almost a century and had an emission spectrum in which the long wavelength component (600–700 nm, red light) was prevalent [6]. Light-emitting diode (LED) technology lamps have a greater energy efficiency and recently replaced incandescent lamps. LED lamps, especially the cold light ones, have an emission spectrum with a considerably short wavelength component (400–500 nm, blue light) [6]. In addition, the increasingly common use of electronic portable devices is contributing to a further increase in the total amount of the blue light to which humans are exposed daily [7,8,9].

The biological effects of light radiation belonging to the visible spectrum were studied especially on retinal cells for both blue and red light [10,11,12,13]. Red light has neuroprotective effects that have been demonstrated in several models of retinal disease [14]. Blue radiation belongs to the area of the spectrum close to that of UV rays and can cause a series of alterations and irreversible damage to eye tissue, including age-related macular degeneration [15,16,17]. Blue light seems to be responsible for harmful biological effects not only on the retina but also on human health, including the immune system and skin [18,19,20]. The skin and eye are the only organs of the human body exposed directly to light radiation. The skin acts both as an organ of separation and defense from the external environment, and it is the communication point between the environment and the individual. In the skin, blue light induces mediators of skin aging, DNA damage and apoptosis in both normal and cancer cells [7,21,22,23]. Regulation of the redox state and the ability to escape cell death or DNA damage is associated with the activation of specific signaling pathways [24,25]. The key molecular elements implicated in skin cells’ response to light have not yet been identified.

This study investigated the alterations in cell viability, apoptotic effects and cell cycle distribution induced by exposure to low- and high-irradiance blue or red light. The proteomic profile was investigated in cells exposed to high-irradiance blue or red light. The light exposure modified molecular key factors that are related to morphological and functional differences between exposed and unexposed cells.

## 2. Results

### 2.1. High-Irradiance Blue or Red Light Reduced HaCaT Cell Viability

**Figure 1:**
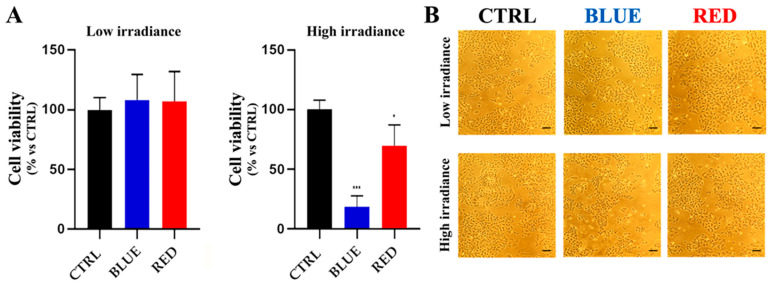
Cell viability and cellular density of HaCaT cells after blue or red light exposure at low or high irradiance. HaCaT cells showed a significant cell viability reduction after exposure to high-irradiance blue or red light (*** = *p* < 0.001, * = *p* < 0.05) (**A**). Optical microscope images confirmed that high-irradiance blue light was able to reduce HaCaT cell density (**B**). Scale bar = 80 µm.

HaCaT cells exposed to low-irradiance blue (BLUE) or red (RED) light sources for 12 *h* exhibited no significant changes in cell viability compared to the control group (CTRL) (Figure 1A). Conversely, exposure to high-irradiance blue or red light decreased HaCaT cell viability by 80% and 30%, respectively, compared to CTRL (Figure 1A). An optical microscope image analysis confirmed that low-intensity blue or red light had no impact on HaCaT cell density (Figure 1B), whereas high-intensity blue light significantly decreased HaCaT cell density compared to the control (Figure 1B).

### 2.2. Apoptotic Effects, Cell Cycle Dysfunction and Alteration in Circadian Clock Gene Expression of High-Irradiance Blue Light Exposure in HaCaT Cells

**Figure 2:**
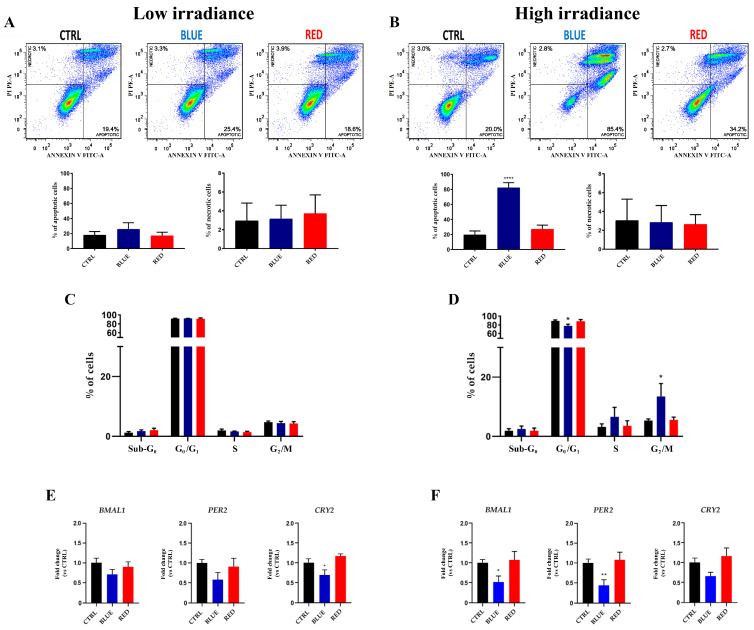
Apoptosis, cell cycle dysfunction and alteration in circadian clock gene expression after low- and high-irradiance blue or red light. Low-irradiance blue or red light did not activate cell death mechanisms (necrosis and/or apoptosis) in HaCaT cells exposed to low- or high-irradiance blue or red light (**A**). High-intensity blue light was able to induce apoptosis in HaCaT cells (**** = *p* < 0.0001); high-irradiance red light could not induce cell death in these cells (**B**). Low-irradiance blue or red light did not alter the cell cycle progression (**C**). The cell cycle progression was arrested in the G_2_/M phase by high-irradiance blue light, which also reduced the percentage of cells in the G_0_/G_1_ phase (* = *p* < 0.05) (**D**). The *CRY2* clock gene was downregulated after low-irradiance blue light exposure (* = *p* < 0.05) (**E**). High-irradiance blue light exposure decreased the *BMAL1* and *PER2* clock genes (* = *p* < 0.05 and ** = *p* < 0.01, respectively) (**F**).

Low-irradiance blue or red light did not induce apoptosis and/or necrosis in HaCaT cells (Figure 2A), whereas high-irradiance blue light induced a significant pro-apoptotic effect (80.2%) in HaCaT cells compared with the CTRL (Figure 2B). Both necrotic and apoptotic cell death mechanisms were not activated by high-irradiance red light (Figure 2B).

Low-irradiance 12 *h* blue or red light treatment did not significantly modify the HaCaT cell cycle distribution compared with the CTRL (Figure 2C). However, high-irradiance blue light was able to induce a significant decrease in cells in the G_0_/G_1_ phase and an increase in the G_2_/M phase in exposed cells compared with the CTRL (Figure 2D).

Since alteration in the circadian clock gene expression in human cells was correlated with cell cycle dysfunction and induction of apoptosis [26], we investigated the expression of *BMAL1*, *PER2* and *CRY2* clock genes. Low-irradiance blue light was able to induce a significant downregulation of the *CRY2* clock gene expression level compared with the CTRL (Figure 2E). In addition, HaCaT cells treated with high-irradiance blue light showed a significant downregulation of the *BMAL1* and *PER2* clock gene levels compared with the CTRL (Figure 2F).

### 2.3. Proteomic Profile Revealed a Role of Oxidative Stress Response-Related Proteins Deregulated in HaCaT Cells Exposed to High-Irradiance Blue Light

**Figure 3:**
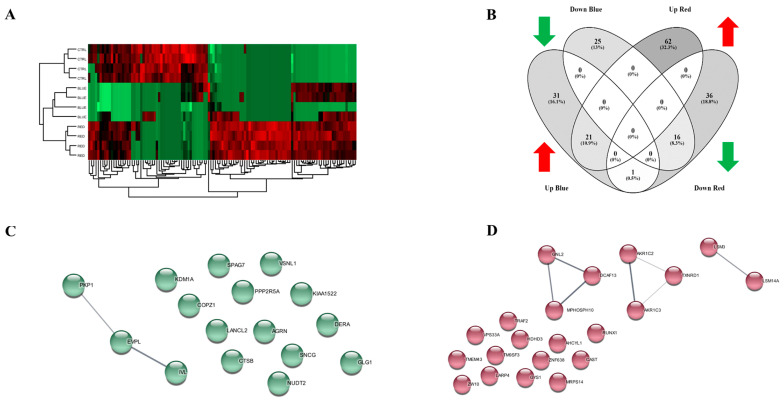
Proteomic profile of HaCaT cells exposed to high-irradiance blue or red light. High-irradiance blue or red light altered the proteome of the HaCaT cells compared with the control cells as shown using hierarchical heat map clusters (**A**). The Venn diagram shows that there were 31, 25, 62 and 36 proteins expressed only in “Up Blue”, “Down Blue”, “Up Red” and “Down Red”, respectively. A total of 16 common proteins were expressed in both “Down Blue” and “Down Red”; 21 common proteins were identified as upregulated after exposure to high-irradiance blue or red light. (**B**). A STRING analysis reported by Cytoscape revealed the correlations between proteins commonly downregulated (**C**) and/or upregulated (**D**) in HaCaT cells exposed to blue or red light.

Proteomic analysis showed a significant deregulation of the proteome in HaCaT cells exposed to high-intensity blue or red light. Hierarchical cluster analysis of the protein expressions in all sample groups demonstrated a clear separation of the treated samples from the controls (Figure 3A). Compared with unexposed cells, HaCaT cells exposed to blue or red light modified their protein profile by altering the expression levels of many proteins (Figure 3B). The data analysis showed that 31 and 62 proteins were exclusively overexpressed in blue and red light, respectively, while 25 and 36 proteins were exclusively underexpressed in blue and red light, respectively (Figure 3B). Additionally, we identified 16 common proteins expressed in both “Down Blue” and “Down Red” (Figure 3B,C and Table 1) and 21 common proteins expressed in both “Up Blue” and “Up Red” (Figure 3B,D and Table 1). The Tenascin C (TNC) protein was the only one protein shared between “Up Blue” and “Down Red”. Interestingly, the proteins Aldo-keto reductase family 1 member C3 (3-alpha hydroxysteroid dehydrogenase, type II) (AKR1C3) and Cytoplasmic thioredoxin reductase 1 (TXNRD1) belong to the 21 common proteins expressed in “Up Blue” and “Up Red” (Figure 3D and Table 1). In addition, the proteomic data analysis indicated that superoxide dismutase type 1 (SOD1) was one of the most upregulated proteins in the HaCaT cells after high-irradiance blue light exposure. The literature data reported that AKR1C3, TXNRD1 and SOD1 are included in the signaling pathways involved in oxidative stress and the cellular response to stress [27,28,29].

### 2.4. High-Irradiance Blue Light Upregulated AKR1C3 and TXNRD1 Expression Levels while Blue or Red Light Exposure Increased the SOD1 Protein Expression Level

**Figure 4:**
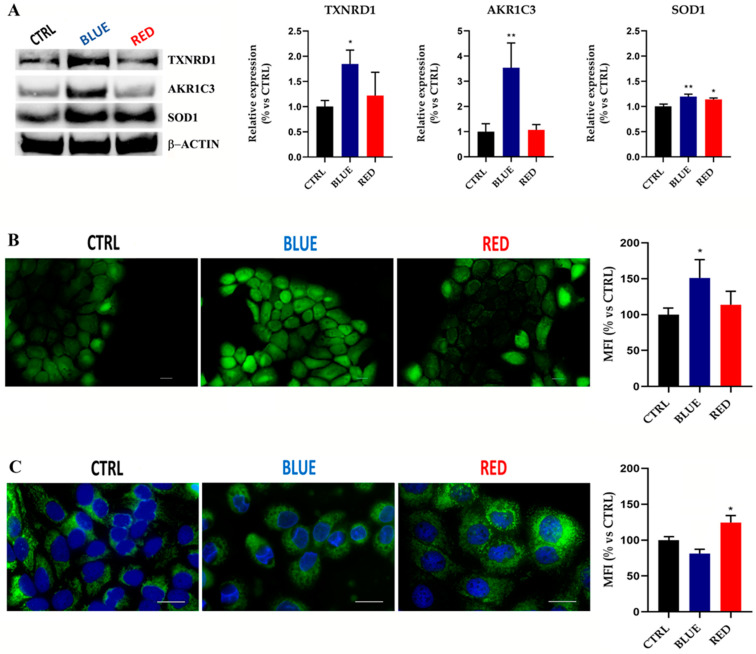
High-irradiance blue or red light exposure affected the TXNRD1, AKR1C3 and SOD1 expression levels and altered the ROS production level and mitochondrial density in HaCaT cells. Western blot analysis showed a significant upregulation of TXNRD1 and AKR1C3 expression levels after high-irradiance blue light exposure (* = *p* < 0.05 and ** = *p* < 0.01, respectively). SOD1 increased its level of expression after exposure to high-irradiance blue or red light (** = *p* < 0.01 and * = *p* < 0.05, respectively) (**A**). The intracellular ROS level increased after high-irradiance blue light exposure (* = *p* < 0.05). Magnification = 200× (**B**). The mitochondrial density increased after high-irradiance red light treatment (* = *p* < 0.05). Magnification = 400× (**C**). Scale bar = 20 µm.

Western blot analysis was used to confirm the proteomic profiling results obtained from the HaCaT cells exposed to high-irradiance blue or red light. TXNRD1 and AKR1C3 were highly expressed in cells exposed to high-irradiance blue light compared with the control group (Figure 4A). On the contrary, high-irradiance red light did not significantly modify the expression level of the TXNRD1 and AKR1C3 proteins (Figure 4A). Moreover, SOD1 was upregulated in the HaCaT cells after high-irradiance blue or red light exposure compared to the CTRL (Figure 4A).

### 2.5. High-Irradiance Blue Light Increased ROS Production Level and Correlated with Higher Mitochondrial Density in HaCaT Cells

Cells exposed to high-irradiance blue light showed a significant increase in their intracellular ROS level compared to the control group (Figure 4B), while in the opposite way, ROS generation was not affected by treatment with high-irradiance red light (Figure 4B). A MitoTracker assay showed a higher mitochondrial density in HaCaT cells exposed to high-irradiance red light compared with the unexposed HaCaT cells (Figure 4C). No alterations in the mitochondrial density in the HaCaT cells exposed to high-irradiance blue light were observed (Figure 4C).

### 2.6. Effect of High-Irradiance Blue or Red Light Exposure on the Cell Structure of HaCaT Cells

**Figure 5:**
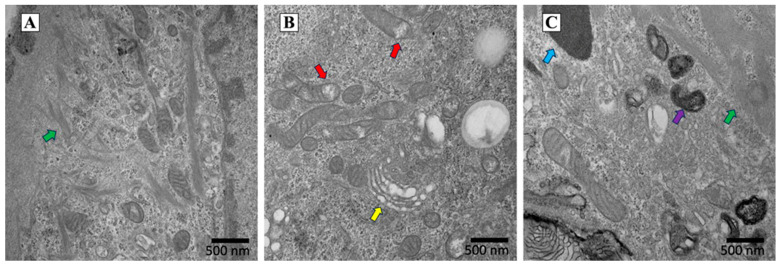
Characterization of HaCaT cell structure after high-irradiance blue or red light exposure using a Transmission Electron Microscope (TEM). The CTRL keratinocytes cells were poorly differentiated and contained tonofilaments (green arrow) (**A**). Blue light exposure had a negative impact on HaCaT cells: the yellow arrow points to an area with a dilated Golgi, while the red arrows identify the presence of damaged mitochondria (**B**). HaCaT cells exposed to red light showed a large amount of tonofilaments (green arrow) and increased lysosomal content (purple arrow). The cyan arrow points to a melanin granule. (**C**). Scale bars are reported in the figures.

The CTRL cells were scarcely differentiated, and their mitochondria were long-branched and hyperfused (Figure 5A). The CTRL cells had more keratohyalin deposits and tonofilaments than the ones exposed to blue light (Figure 5A). The cells exposed to blue light (BLUE) showed signs of damage: many cells were vacuolated and had damaged mitochondria and a dilated Golgi (Figure 5B). The cells exposed to red light contained a large amount of tonofilaments and showed a higher lysosomal content with respect to the BLUE and CTRL conditions (Figure 5C).

## 3. Discussion

The present study showed that high-irradiance blue or red light acted by stimulating the synthesis of proteins belonging to different signaling pathways involved in cell protection, cell death and cell damage. These responses are probably related to an increase in cellular ROS production after light exposure [30,31]. On the other hand, our results showed that low-irradiance blue or red light did not affect the cell viability or cell cycle program and did not induce cell apoptosis or necrosis in the HaCaT cells. The low-irradiance blue light exposure induced only a downregulation of the *CRY2* clock genes in keratinocytes. Cryptochrome 2 (CRY2), one of the circadian clock proteins, was implicated in DNA damage checkpoint control, regulating important cell cycle progression genes and playing a pivotal role in the control of the cell proliferation rate [32,33].

High-irradiance blue or red light decreased the cell viability and led to different cellular responses in keratinocytes. The keratinocytes lost cell viability under the stimulus of high-irradiance blue light. In these cells, G_2_/M cell cycle arrest was induced, and the apoptotic rate increased. At the same time, high-irradiance blue light decreased the expression levels of the *BMAL1* and *PER2* clock genes in the HaCaT cells, suggesting a circadian rhythm dysregulation [34,35,36]. High-irradiance red light did not induce cell death and did not alter the clock gene expression levels. Indeed, it was confirmed that the effects due to high-irradiance blue light exposure in the HaCaT cells were more toxic than those of high-irradiance red light [23]. The toxic effect of blue light on keratinocytes can be prevented by chemicals acting on inflammation and oxidative stress [37]. Among them, some natural compounds were promising agents for skin damage prevention [38]. Specifically, zeaxanthin is a carotenoid compound belonging to the family of xanthophyll that can protect human cells by blocking a wide variety of inflammation-related factors activated by exposure to blue light [39].

The cellular responses after exposure to high-irradiance blue or red light are related to several molecular mechanisms. Keratinocytes exposed to high-irradiance blue light, modified their proteomic profile compared with both the CTRL cells and those exposed to high-irradiance red light. As reported in the Venn diagram (Figure 3B), only the TNC was differently expressed in HaCaT cells exposed to high-irradiance blue or red light; its expression level increased after exposure to high-irradiance blue light and decreased after high-irradiance red light. The effect induced by high-irradiance blue light in the HaCaT cells probably induced the expression of Tenascin C, which has a key role in inflammatory and fibrotic mechanisms [40]. Interestingly, the proteomic profile revealed that 16 and 21 common genes were down- and upregulated, respectively, in the HaCaT cells exposed to high-irradiance blue or red light. AKR1C3 and TXNRD1 were 2 of the 21 common genes upregulated in HaCaT cells after high-irradiance blue or red light exposure. These two proteins, as predicted by STRING, belong to the “Ferropoptosis” WikiPathway (WP4313) [41]. Ferroptosis is an iron-dependent oxidative programmed cell death in which reactive oxygen species (ROS) accumulation plays a pivotal role [42,43]. Western blot analysis confirmed that high-irradiance blue light induced an upregulation of AKR1C3 and TXNRD1 in the HaCaT cells. This correlated with an increased level of ROS production after blue light exposure. Differently, high-irradiance red light did not significantly modify the AKR1C3 and TXNRD1 protein expression levels in the HaCaT cells. The results also showed that red light had no effect on the intracellular levels of ROS production, although it did stimulate an increase in the mitochondrial density. The Western blot results showed that SOD1 was upregulated in both the high-irradiance blue- and red-light-treated HaCaT cells. SOD1 has been found to be a primary regulator of redox signaling in diverse physiological and pathological processes [44,45]. We hypothesized that the stimulus of high-irradiance blue or red light induced an increase in reactive oxygen species (ROS) that correlated with mitochondrial alterations.

Different molecular mechanisms probably correlated with exposure to high-irradiance blue vs. red light. Effectively, as shown in the Transmission Electron Microscope (TEM) images of HaCaT cells exposed to high-irradiance blue or red light, these treatments differently modified the cell ultrastructure. High-irradiance blue light caused mitochondrial alteration, dilated the Golgi complex and caused cellular damage. The HaCaT cells exposed to high-irradiance red light showed morphological characteristics related to keratinocyte maturation processes leading to a differentiated state.

## 4. Materials and Methods

### 4.1. Cell Culture

The HaCaT cells were obtained from the Experimental Zooprophylactic Institute of Lombardia and Emilia Romagna (Brescia, Italy). The cells were cultured in DMEM High Glucose (Euroclone, Milan, Italy) supplemented with 10% fetal bovine serum (Euroclone), 1% penicillin/streptomycin (Euroclone) and 1% glutamine (Euroclone) in a 5% CO_2_ humidified atmosphere at 37 °C and subcultured twice a week.

### 4.2. Light Exposure

High-power blue and red single-color LEDs (LD W5AM and LH W5AM Golden DRAGON**^®^** Plus, respectively; Osram, Munich, Germany) were used as the light sources. The LED viewing angle was 170°, and the cells were placed 14 cm above the light sources. The homogenous distribution of light and the spectrum of emission of each monochromatic LED were verified using an illuminance meter with a spectral sensor (CL-70; Konica Minolta Sensing, Inc., Chiyoda-ku, Tokyo, Japan). The dominant wavelength was 465 nm for blue and 658 nm for red LEDs. The irradiance at peak wavelength at the cell surface was 0.84 W/m^2^ for blue and 1.10 W/m^2^ for red LEDs for high intensity and 0.01 W/m^2^ for both blue and red LEDs for low irradiance. The irradiance corresponded to the same total spectrum irradiance of 28.50 W/m^2^ and 0.28 W/m^2^ for both light sources at high and low irradiance, respectively. The light energy transferred to the cells every day was 1.23 J/mm^2^ for high irradiance and 0.01 J/mm^2^ for low irradiance. The light exposure was set to reproduce outdoor light (high irradiance) and indoor light (low irradiance). Blue and red light were specifically chosen to test the opposite sides of the spectrum of visible light. Constant darkness was considered the sham light source (control cells (CTRL)).

Cells were starved in DMEM low glucose (Euroclone) supplemented with 0.1% FBS (Euroclone) and 1% penicillin/streptomycin (Euroclone) for 24 h. Before the light exposure, the culture medium was replaced with DMEM without phenol red (Lonza, Basel, Switzerland) supplemented with 0.1% FBS (Euroclone) and 1% penicillin/streptomycin (Euroclone). The HaCaT cells were exposed in a 12 h light/dark cycle (12L:12D) at high and low irradiance to sham, blue or red light in the incubator at 37 °C and 5% CO_2_ for 3 days.

The LEDs in the incubator were fixed on an aluminum tank with thermal conductive paste, and the water circulation inside the tank (Amersham Multitemp III; GE Healthcare, Chicago, IL, USA) extracted the heat generated by the LEDs so they could work at a constant temperature. These conditions assured a constant electric current and therefore a constant emitted energy. The incubator was divided into three light zones: constant darkness, blue and red zones. Interference between light sources was prevented by using black curtains, and sham exposure was additionally ensured by wrapping the plate with aluminum foil. The HaCaT cells were seeded in 6, 12 or 96 well-plates and positioned in the incubator 12 cm over the sham or light sources. In this way, cells on the bottom of the plates were directly irradiated by light. Phenol-free medium prevented the influence of the medium on the light-exposure characteristics. To avoid the stray light interference between wells, three different plates were used in each experiment (i.e., one for each light zone). Air circulation inside the incubator was ensured using a fan. To exclude any thermal effects, the temperature at the cell level was verified and constantly measured during experiments with a Thermochron iButton DS1922L (Maxim Integrated Products, San Jose, CA, USA).

### 4.3. Cell Viability Assay

To analyze the cell viability after the light exposure, a Cell Proliferation Kit II XTT (Merck KGaA, Darmstadt, Germany) was used according to the manufacturer’s protocol. Briefly, 1.6 × 10^4^ cells per well were seeded in 96-well plates (Costar, Corning, NY, USA) and exposed to blue or red LED light (constant darkness was used as a control). Then, 50 µL of XTT solution was added to each well, followed by incubation for 4 h at 37 °C and 5% CO_2_. The absorbance at 450 nm (with 650 nm as the reference wavelength) was measured using an absorbance microplate ELISA plate reader (Sunrise; Tecan Group Ltd., Männedorf, Switzerland). The proliferation index was expressed as the relative change with respect to the controls set as 100%. Each assay was done in triplicate.

### 4.4. Cell Density Assay

Cells were seeded at a density of 5 × 10^4^/mL in 12-well plates (Costar, Corning) and exposed to blue or red light. The cell density was analyzed under a microscope (Nikon Eclipse Ti2 Inverted Microscope; Nikon, Tokyo, Japan). The assay was performed in triplicate.

### 4.5. Apoptosis Assay

A total of 2 × 10^5^ cells/mL of HaCaT cells were seeded in 6-well plates (Costar, Corning) for each well and exposed to light. Cellular apoptosis was detected using an Annexin V/Propidium Iodide (PI) apoptosis detection kit (eBioscience™ Annexin V-FITC Apoptosis Detection Kit; Thermo Fisher, Milan, Italy) according to the manufacturer’s instructions. Flow cytometric analysis was performed using a FACSCalibur™ flow cytometer (BD Biosciences, San Jose, CA, USA) equipped with CellQuest version 5.3 software set on a linear scale (BD Pharmingen, Franklin Lakes, NJ, USA). The data were analyzed using FlowJo™ Software (FlowJo™ Software for Windows, Version 7.6.1, BD Company, Ashland, OR, USA). The assay was performed in triplicate.

### 4.6. Cell Cycle Analysis

The cell cycle analysis of cells exposed to light and the control cells was performed using propidium iodide (PI) staining (Merck KGaA, Darmstadt, Germany). After light exposure, the cells were harvested and centrifuged at 300× g for 6 min at room temperature, fixed with 4.5 mL of cold ethanol solution (70% in PBS) and kept on ice at 4 °C for at least 2 h. Subsequently, the cells were washed twice with PBS and resuspended in PI staining solution with 0.1% TritonX-100 (Santa Cruz Biotechnology, Inc.; Dallas, TX, USA), 0.2 mg/mL of RNAse (Merck KGaA) and 2 mg/mL of PI (Merck KGaA). The cells were incubated at 37 °C for 15 min. The flow cytometric analysis was performed using a FACSCalibur™ flow cytometer (BD Biosciences). The data were analyzed using FlowJo™ Software (FlowJo™ Software for Windows, Version 7.6.1.). A total of 20,000 cells was acquired for each well. The assay was performed in triplicate.

### 4.7. Total RNA Extraction and Quantitative Real-Time PCR (qRT-PCR)

The total RNA was isolated from the HaCaT cells after light exposure using a Total RNA Isolation kit (Norgen Biotek, Thorold, ON, Canada) following the manufacturer’s protocol. Quantification of the RNA was performed with a Nanodrop (NanoDrop 1000, Thermo Fisher, Waltham, MA, USA), and the total RNA was stored at −80 °C until use. One microgram of total RNA was reverse-transcribed using a Prime Script RT Reagent Kit with the gDNA Eraser (Takara, Göteborg, Sweden) according to the manufacturer’s instructions. Quantitative real-time PCR (qRT-PCR) was conducted using a QuantStudio™ 1 Real-Time PCR System (Thermo Fisher). The amplification of human *BMAL1*, *PER2*, *CRY2* and *GAPDH* (used as reference) genes was performed using a TaqMan primer and probe set (Thermo Fisher) and Fluocycle II Master Mix for the probes (Euroclone). The relative quantity of mRNA specific to each of the target genes was calculated using the 2^−ΔΔCT^ method: ΔΔCt = ΔCt (CTRL) − ΔCt (cells exposed to blue or red light). The experiments were performed in triplicate and were repeated three times.

### 4.8. Protein Extraction and Digestion for MS Analysis

A total of 2 × 10^5^ cells/well of HaCaT cells were seeded in 6-well plates (Costar, Corning) and exposed to high-irradiance light. After light exposure, the cells were washed with 1× PBS (Euroclone), and urea buffer (100 mM of Tris HCl (pH 8.5) and 8 M of urea; Merck KGaA) was used to scrape the cells. Lysates were incubated on ice, and cell lysis was promoted via vortex every 10 min. The samples were centrifuged (Eppendorf**^®^** Microcentrifuge 5415; Merck KGaA) at 16,000× *g* for 20 min at 4 °C, and protein concentrations were measured using a Bradford assay (Merck KGaA). Next, 50 µg of protein was reduced with TCEP, (Merck KGaA) alkylated with chloroacetamide (Merck KGaA) and digested with Lys-C and trypsin (Merck KGaA), and then the peptides were desalted on a homemade C18 StageTip.

### 4.9. Mass Spectrometry Analysis

A total of 4 µL of each sample was analyzed on a LC–ESI–MSMS quadrupole Orbitrap Q Exactive-HF mass spectrometer (Thermo Fisher). Separation of the peptides was performed on a linear gradient from 95% solvent A (2% ACN and 0.1% formic acid; Merck KGaA) to 30% solvent B (80% acetonitrile and 0.1% formic acid; Merck KGaA) for 210 min, then from 30% solvent B in 20 min to 100% solvent B in 2 min at a constant flow rate of 0.25 µL per minute on a UHPLC Easy-Nlc 1000 (Thermo Fisher) connected to a 23 cm fused-silica emitter with a 75 µm inner diameter (New Objective, Inc. Woburn, MA, USA) packed in-house with ReproSil-Pur C18-AQ 1.9 µm beads (Dr Maisch Gmbh, Ammerbuch, Germany) using a high-pressure bomb loader (Proxeon, Odense, Denmark). Mass spectrometry (MS) data were acquired using a data-dependent top 20 method for HCD fragmentation. The full-scan MS spectra (300–1650 Th) were acquired in the Orbitrap with a resolution of 60,000, an AGC target of 3 × 10^6^ and an IT of 20 ms. For the HCD spectra, the resolution was set to 15,000 at *m*/*z* 200, the AGC target to 1 × 10^5^, the IT to 80 ms, the NCE to 28% and the isolation width to 2.0 *m*/*z*.

The raw data were processed via MaxQuant version 1.5.2.8. Peptides were identified from the MS–MS spectra searched against the Uniprot_cp_Homo Sapiens (98,036 entries) using the Andromeda search engine, in which trypsin specificity was used with up to two missed cleavages allowed. Cysteine carbamidomethylation was used as a fixed modification and methionine oxidation and protein amino-terminal acetylation as variable modifications. The mass deviations for the MS and MS–MS peaks were set at 5 and 20 ppm, respectively. The peptide and protein false discovery rates (FDRs) were set to 0.01, the minimal length required for a peptide was six amino acids, and a minimum of two peptides and at least one unique peptide were required for high-confidence protein identification. The lists of identified proteins were filtered to eliminate reverse hits and known contaminants. Label-free analysis was carried out, including a ‘match between runs’ option (time window of 5 min). A minimum ratio count of two was considered, and the ‘LFQ53 intensities’, which were the intensity values normalized across the entire data set, were used.

Statistical analyses were conducted using the Perseus program (Version 1.5.1.6) in the MaxQuant environment with a *p*-value of 0.05. Missing values were replaced with random numbers drawn from a normal distribution using the function ‘imputation’ (with a width of 0.3 and a downshift of 1.8 (separately for each column)). Hierarchical clustering was set using the following parameters: distance, Euclidean, linkage, average, number of clusters and 300 (for both row and column tree dendrograms generated by clustering in the Perseus environment).

### 4.10. Bioinformatics Analysis

Each differentially expressed protein was assigned to the respective human official NCBI Gene Symbol identifier and was analyzed using the Enrichr (http://amp.pharm.mssm.edu/enrichr, accessed on 13 December 2020) web tool. Statistical enrichment of Gene Ontology (GO) (sections “Biological Process”, “Molecular Function” and “Cellular Component” (release 2018)) using the Kyoto Encyclopedia of Genes and Genomes (KEGG, 2019) pathway database and WikiPathways 2019 were assessed by submitting the gene lists to the Enrichr tool. Up- and downregulated genes were separately submitted to the web tools. Statistically significant terms were selected for further analyses if an adjusted *p*-value < 0.05 was reached according to the Benjamini–Hochberg (BH) method for correction for multiple hypotheses testing. The protein–protein interaction (PPI) networks were built with the Search Tool for the Retrieval of Inter-acting Genes/Proteins (STRING, https://string-db.org/, accessed on 3 February 2023) and Cytoscape (software version 3.9.1) [46].

### 4.11. Protein Extraction and Western Blotting

The pellets of HaCaT cells exposed to high-irradiance blue or red light were resuspended in Radioimmunoprecipitation Assay buffer (RIPA buffer, 50 mM of Tris HCl (pH 7.4), 1% NP 40, 0.1% SDS, 150 mM of NaCl and 2 mM EDTA; Merck KGaA) containing the protease inhibitor cocktail (Roche Applied Science, Indianapolis, IN, USA) that was used for protein extraction. The protein concentration was determined using Bradford reagent (Merck KGaA). The total protein extracts (30 μg) were separated via 4–12% sodium dodecyl sulfate-polyacrylamide gel electrophoresis (Bolt 4–12% Bis-Tris Plus; Thermo Fisher) in MES SDS running buffer (Thermo Fisher) for 1 h at 200 V. A protein ladder (SeeBlue**^®^** Plus2 Prestained, Thermo Fisher) was used as a reference for protein size (3–198 kDa). Proteins were transferred onto a nitrocellulose membrane (NC; Nitrocellulose Blotting Membrane GE Healthcare Life Sciences, Amersham™ Protran™). Membranes were blocked in Tris-buffered saline with 0.1% Tween 20 (TBS-T) containing 5% fat-free dry milk for 60 min and then incubated overnight at 4 °C with primary mouse monoclonal against Aldo-keto reductase family 1 member C3 (anti-AKR1C3, 1:5000; Merck KGaA), primary rabbit polyclonal against Thioredoxin reductase 1 (anti-TXNRD1, 1:1000; Merck KGaA, Darmstadt, Germany) and superoxide dismutase 1 (anti-SOD1, 1:1000; Merck KGaA). Primary rabbit polyclonal anti-human anti-GAPDH antibody (1:1000; Bethyl Laboratoires Inc., Montgomery, TX, USA) was used as the loading control. Membranes were washed with PBS-0.1% Tween and incubated for 1 h at room temperature with horseradish peroxidase-linked secondary antibodies, Goat anti-rabbit IgG (H + I) peroxidase/HRP-conjugated (1:3000; Elabscience**^®^** Biotechnology, Huston, TX, USA) or anti-mouse IgG (1:10,000) (Goat anti-mouse IgG (H + I) HRP-conjugated; Bethyl Laboratoires). An Alliance Mini (UVITEC Cambridge, Cambridge, UK) system was used for analyzing the Western blot results. Blot bands were quantified with UVITEC software (Nine Alliance Mini Software, version 17.02), and their intensity was normalized via comparison to the housekeeping protein GAPDH. The intensity of each band was compared to the negative controls, and any change was expressed as a percentage.

### 4.12. Intracellular ROS Detection

Intracellular ROS levels were investigated using the fluorescent dye 2′7′-dichlorofluorescein diacetate (DCFDA; oxidized with hydrogen peroxide to DCF, Merck KGaA). The HaCaT cells were seeded on a cover glass in six-well plates (Costar, Corning, NY, USA) at a density of 2 × 10^5^ cells/mL. After light treatments, the cells were washed twice with PBS (Euroclone), and DCFH-DA solution in serum-free medium was added at a concentration of 10 µM per well for 45 min. The slides were washed 3 times, and the fluorescence was measured using a fluorescence microscope (Nikon Eclipse Ti2 Inverted Microscope; Nikon, Tokyo, Japan). The mean fluorescence intensity (MFI) from four random fields/slides was analyzed using ImageJ 1.41 software (National Institutes of Health, Bethesda, MD, USA). The mean fluorescence intensity (MFI) of the DCFH-DA was used to indicate the amount of ROS. The analyses were performed in triplicate.

### 4.13. Mitotracker Green Staining

Mitochondria were stained using FITC-conjugated Mitotracker green fluorescent probe (Thermo Fisher). The HaCaT cells were seeded on 5 × 104 cells/mL chamber slides (Nunc Inc., Toronto, ON, Canada). After light treatments, the cells were washed with DMEM free of red phenol (Lonza) and FBS. Then, 500 uL of 100 nM prewarmed (37 °C) probe was added and then incubated at 37 °C in the incubator for 45 min. Cell nuclei were stained with Hoechst (Merck KGaA). Cells were observed with a fluorescence microscope (Nikon Eclipse Ti2 Inverted Microscope (Nikon)). The mean fluorescence intensity (MFI) from four random fields/slides was analyzed using ImageJ 1.41 software. The staining reactions were performed in triplicate.

### 4.14. Transmission Electron Microscope (TEM)

The cells were plated on ACLAR and cultured at 1 × 10^5^ cells/cm^2^ (Ted Pella, Redding, CA, USA); chips were trimmed to fit into the 6-well plates. After light treatments, the cells were fixed with glutaraldehyde 2%, 0.7% tannic acid and 30 mM sucrose in 0.1 M of cacodylate buffer (pH 7.0) for 1 h at room temperature and subsequently overnight at 4 °C. Cells were post-fixed in 0.5% osmium tetroxide for one additional hour and then dehydrated with an acetone series and flat-embedded in Luft epoxy resin using a polystyrene capsule. After hardening of the resin, the ACLAR was peeled off and ultrathin sections (50 nm) obtained. The sections were stained with uranyl acetate alcoholic solution and lead citrate then imaged at 80 kV in a Philips CM12 electron microscope (Philips, Amsterdam, The Netherlands). The images are representative of three independent experiments.

### 4.15. Statistical Analysis

Three independent experiments were conducted, and the results were reported as the mean ± standard deviation (SD). Graphpad Prism software (GraphPad Prism version 7.00 for Windows, GraphPad Software, La Jolla, CA, USA) was used for all statistical analyses. One-way ANOVA and Dunnett’s multiple comparison test as a post hoc test were used to evaluate the statistical significance for each treatment; *p* < 0.05 was considered statistically significant.

## 5. Conclusions

Blue light and red light had a biological effect on keratinocytes that was different in relation to the intensity of these stimuli. Keratinocytes exposed to low-irradiance blue or red light did not modify their viability and/or cell cycle progression. Keratinocytes exposed to high-irradiance blue or red light altered their cell viability, but only blue light was able to induce apoptosis and impairment of the cell cycle. The proteomic profile was modified in the expression of genes related to oxidative stress response. The present study highlights the need for further investigations of the mechanisms activated by blue or red lights on human keratinocytes. Our results can be useful to properly address the use of light as a therapeutic option and to develop appropriate preventive strategies for skin damage.

## Figures and Tables

**Table 1:** The 16 common proteins downregulated and 21 common proteins upregulated in the HaCaT cells after high-irradiance blue or red light exposure.

16 COMMON PROTEINS DOWNREGULATED	21 COMMON PROTEINS UPREGULATED
AGRN	AHCYL1
COPZ1	AKR1C2
CTSB	**AKR1C3**
DERA	CAST
EVPL	DCAF13
GLG1	GNL2
IVL	GYS1
KDM1A	HDHD3
KIAA1522	LARP4
LANCL2	LSM14A
NUDT2	LSM3
PKP1	MPHOSPH10
PPP2R5A	MRPS14
	RUNX1
SNCG	TM9SF3
SPAG7	TMEM43
VSNL1	TRAF2
	**TXNRD1**
	VPS33A
	ZNF638
	ZW10

## Data Availability

The raw proteomic data have been uploaded to the jPOST repository with the identifiers JPST002058 (jPOST) and PXD046776 (ProteomeXchange).
